# Carfilzomib-Associated Thrombotic Microangiopathy in a Multiple Myeloma Patient: Complement Genetic Alterations Impact and Eculizumab Therapy

**DOI:** 10.7759/cureus.89189

**Published:** 2025-08-01

**Authors:** Joana Lopes, Vítor Fernandes, Nuria Paulo, Pedro Lisboa Gonçalves, Rafael Figueiredo, Luis Mendonça, Bernardo Faria, Ricardo Neto, Rui Bergantim, João Frazão

**Affiliations:** 1 Nephrology Department, Unidade Local de Saúde São João, Porto, PRT; 2 Hematology Department, Unidade Local de Saúde São João, Porto, PRT

**Keywords:** acute kidney injury, carfilzomib, eculizumab, multiple myeloma, thrombotic microangiopathy

## Abstract

Carfilzomib is a second-generation proteasome inhibitor used in relapsed or refractory multiple myeloma (MM). Although effective, it can be associated with rare but life-threatening complications. We describe a 73-year-old woman with relapsed IgG-kappa MM who developed thrombotic microangiopathy (TMA) and acute kidney injury (AKI) requiring hemodialysis, three weeks after initiating a carfilzomib-based regimen. Genetic testing showed a homozygous CFHR3-CFHR1 deletion and variants of uncertain significance in the CFI and ADAMTS13 genes. The patient underwent plasmapheresis followed by treatment with eculizumab, resulting in full renal recovery. This case highlights a rare but potentially reversible complication of carfilzomib therapy, likely driven by complement activation and endothelial injury. It also underscores the role of genetic predisposition and the therapeutic benefit of complement inhibition with eculizumab. Early recognition, drug discontinuation, and consideration of both genetic screening and complement inhibition may improve outcomes in at-risk patients.

## Introduction

Carfilzomib is a powerful proteasome inhibitor approved for treating multiple myeloma (MM) across all lines of treatment, including in relapsed or refractory cases, for patients who have not responded to prior therapies, including bortezomib [1,2]. While effective, carfilzomib has been associated with significant adverse effects, including cardiovascular, pulmonary, and renal complications. Among these, thrombotic microangiopathy (TMA) has been reported as a rare but severe complication [3-5].

TMA is a clinicopathologic syndrome defined by the triad of microangiopathic hemolytic anemia, thrombocytopenia, and organ injury due to thrombotic occlusion of small vessels, particularly arterioles and capillaries. The complement system plays a central role in the pathogenesis of several forms of TMA, most notably in complement-mediated TMA. In these cases, dysregulation of the alternative complement pathway, due to genetic mutations or acquired autoantibodies, leads to uncontrolled complement activation on endothelial surfaces. This results in excessive formation of C3b and the terminal complement complex (C5b-9), causing direct endothelial injury, platelet activation, and microvascular thrombosis. Complement dysregulation can also be triggered or unmasked by secondary conditions such as pregnancy, certain drugs, severe hypertension, or transplantation [6-8].

We present a case of a patient with relapsed MM who developed carfilzomib-associated TMA and was later found to have a complement gene variant.

## Case presentation

A 73-year-old Caucasian woman, diagnosed with IgG-kappa MM in February 2020, promptly began treatment with dexamethasone, thalidomide, and bortezomib, achieving a very good partial response, followed by intensification with high-dose melphalan and autologous stem cell transplantation in January 2021 and lenalidomide maintenance therapy. Her disease relapsed in January 2024, prompting the initiation of daratumumab, carfilzomib, and dexamethasone. Her previous medical history included well-controlled type 2 diabetes mellitus and hypertension, with a baseline serum creatinine (sCr) of 0.7 mg/dL. 

Three weeks after initiating carfilzomib, the patient presented to the emergency department with fever (tympanic temperature of 39.1ºC) but no other symptoms. She was hemodynamically stable. Initial blood workup showed worsening anemia and thrombocytopenia, elevated C-reactive protein and lactic dehydrogenase (LDH), along with acute kidney injury (Table 1). Urinalysis revealed erythrocyturia, and renal ultrasound showed normal-sized kidneys with preserved echogenicity, ruling out obstruction. On the second day of hospitalization, the patient became anuric and required urgent start of hemodialysis. At this time, low serum haptoglobin levels (2-3 g/L) and the presence of schistocytes on the peripheral blood smear were both documented. Initially, direct Coombs had a low degree of positivity, which was interpreted to be likely related to recent blood transfusion and possibly a side-effect of daratumumab therapy. Given the presence of microangiopathic hemolysis and renal dysfunction, TMA was strongly suspected. 

**Table 1:** Laboratory parameters one month before and at admission SARS-CoV-2 - severe acute respiratory syndrome coronavirus 2; PCR - polymerase chain reaction; CMV - cytomegalovirus; HBsAg - hepatitis B surface antigen; HCV Ab - hepatitis C virus antibody; HIV - human immunodeficiency virus

Parameter	Reference range		Previous value (1 month earlier)		Value at admission
Hemoglobin (g/dL)	12.0-16.0		10.7		8.6
Leucocytes (x10^9 ^/ µL)	4.00-11.00		4.58		4.96
Plateles (x10^9 ^/ µL)	150-400		121		43
Alanine aminotransferase (U/L)	10-31		17		61
Gamma-glutamyl transferase (U/L)	7-32		14		28
Lactic dehydrogenase (U/L)	135-225		156		1954
Total bilirubin (mg/dL)	<1.20		0.48		0.69
Direct bilirubin (mg/dL)	<0.40		< 0.01		0.13
Creatinine (mg/dL)	0.51-0.95		0.70		2.10
Urea (mg/dL)	10-50		46		168
Uric Acid (mg/dL)	2.3-6.1		4.5		8.2
Sodium (mEq/L)	135-147		134		129
Potassium (mEq/L)	3.5-5.1		4.1		3.9
Phosphorus (mg/dL)	2.7 – 4.5		4.4		5.1
Calcium (mEq/L)	4.1 - 5.2	4.9		3.7	
C-Reactive Protein (mg/L)	<3.0	1.3		156.1	
C3c (mg/dL)	83-177	-		90	
C4 (mg/dL)	12-36	-		13	
SARS-CoV-2 PCR	-	-		Negative	
Respiratory syncytial virus PCR	-	-		Negative	
Influenza virus PCR	-	-		Negative	
Legionella urinary antigen	-	-		Negative	
Pneumococcus urinary antigen	-	-		Negative	
Blood cultures	-	-		Negative	
Urine cultures	-	-		Negative	
CMV PCR	-	-		Negative	
HBsAg	-	-		Negative	
HCV Ab	-	-		Negative	
HIV	-	-		Negative	

Blood and urine cultures, as well as *Legionella* and *Pneumococcus* urinary antigens, and nasopharyngeal viral panels, were all negative. A PLASMIC score <5 indicated a low probability of thrombotic thrombocytopenic purpura (TTP). Subsequent ADAMTS-13analysis confirmed normal activity (63%), effectively excluding TTP. Complement levels (C3 and C4) were within normal range, and the absence of diarrhea made typical hemolytic uremic syndrome (HUS) less likely. Given the recent initiation of carfilzomib and the absence of alternative causes, a diagnosis of severe carfilzomib-associated TMA was made. A renal biopsy was not performed due to severe thrombocytopenia. Blood samples were sent for genetic and functional complement studies.

**Figure 1:**
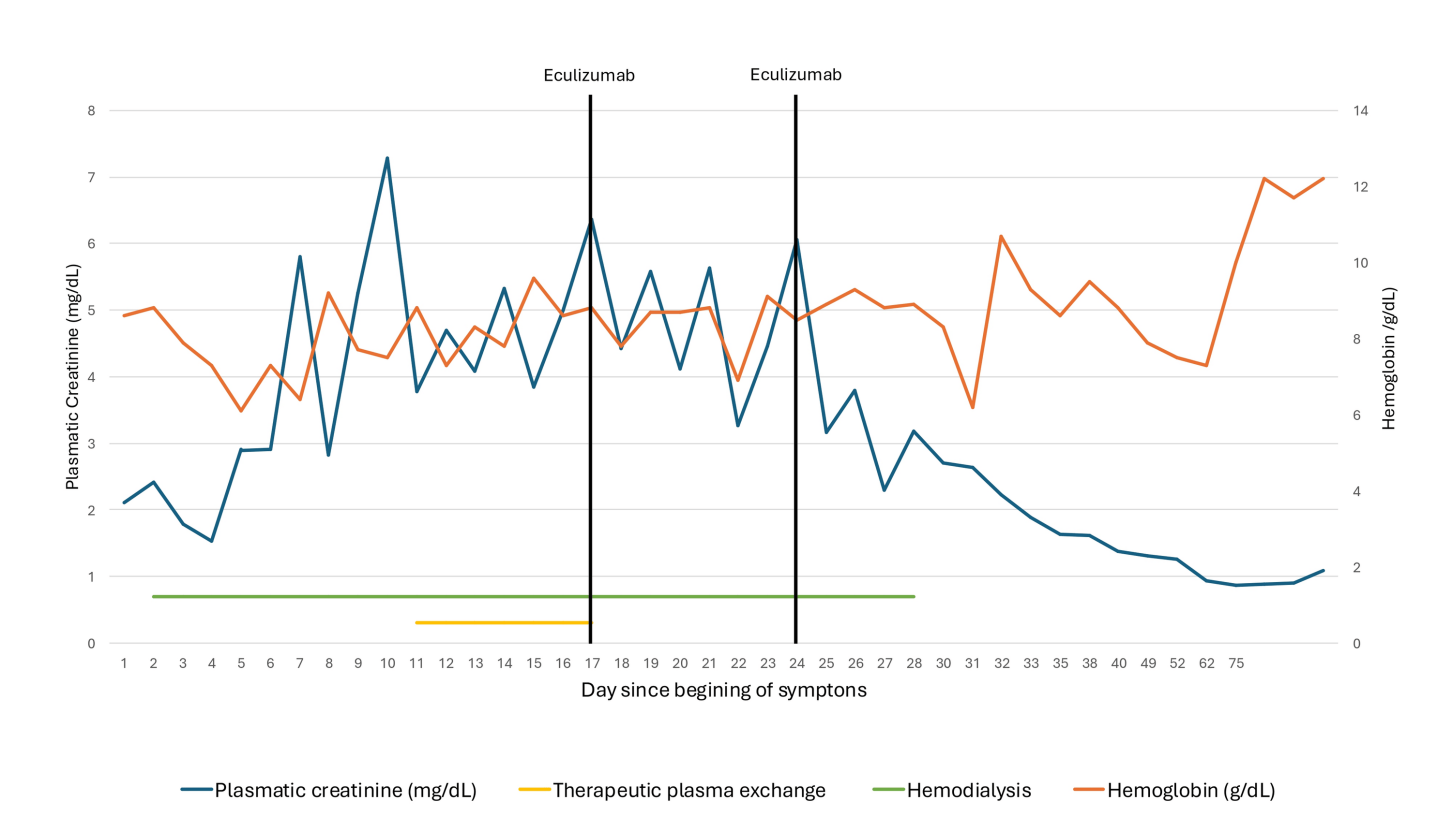
Hemoglobin and creatinine evolution throughout the clinical course

Carfilzomib was discontinued, and the patient underwent daily therapeutic plasma exchange (TPE) for seven days, until eculizumab was approved and administered. Following two doses of eculizumab (administered one week apart), the patient's renal function improved, allowing discontinuation of hemodialysis after a total of 27 days (Figure 1). Treatment with eculizumab was continued with 900 mg weekly for the first four weeks, followed by 1200 mg every two weeks for a total of three months. After this period, renal function normalized, and hematologic parameters showed significant improvement, and eculizumab was stopped. Currently, one year after this event, the patient continues to follow up in the hematology clinic with daratumumab, bortezomib, and dexamethasone as her current therapies, achieving a very good partial response and maintaining normal kidney function with a serum creatinine level of 0.8 mg/dL.

Genetic testing identified a homozygous deletion in the CFHR3-CFHR1 gene, a heterozygous insertion in the CFI gene (variant of uncertain significance), and a heterozygous missense variant in the ADAMTS13* *gene (variant of uncertain significance). 

## Discussion

This case highlights a carfilzomib-associated TMA in an MM patient, after one cycle of treatment with a carfilzomib-containing regimen for relapsed disease. 

Patients with MM have an increased susceptibility to TMA, a risk that is further heightened by treatment with proteasome inhibitors like carfilzomib, as studies indicate this as a side effect in 1-5% of patients treated with this drug [4,9]. Additionality, the homozygous deletion observed in the CFHR3-CFHR1 gene, which has been associated with alternative complement pathway dysregulation, was likely a risk factor contributing to the development of this acute disease. Furthermore, infections have been suggested as a possible "second hit" in drug-induced TMA [4]. Although no evidence of an infectious agent was identified after extensive work-up, our patient did present with fever at admission, raising the clinical hypothesis. This case, therefore, is a good example of the possible interplay between genetic predisposition and other risk factors, such as drugs and infections, in the development of TMA.

The time of onset of carfilzomib-associated TMA varies, with a median reported onset of three months, but a wide range has been observed [3,4]. This variability likely reflects different underlying mechanisms. Early-onset cases may involve direct endothelial toxicity, while later-onset cases could be driven by a classic dose-dependent mechanism [3,10,11]. Carfilzomib has been proposed to reduce vascular endothelial growth factor (VEGF) levels through NF-κB inhibition, potentially leading to glomerular endothelial injury and renal TMA [10]. In this patient, TMA developed rapidly after just one cycle, raising the possibility of direct endothelial toxicity, which may have been exacerbated by the CFHR3-CFHR1 gene deletion [4,12].

Carfilzomib was discontinued, and TPE was used as initial therapy for seven days, pending the availability of eculizumab. No significant improvements in either kidney function or hematologic parameters were noted, as described by others [13]. Eculizumab has been used in carfilzomib-induced TMA, but outcomes are inconsistent [4,5]. In this patient, it was started on day 17 of renal failure, which was later than in most reports. Despite this delay, the patient experienced renal recovery and hematologic improvement after only two doses of eculizumab. 

A renal biopsy remains the gold standard for diagnosing TMA, typically revealing arteriolar and capillary thrombi, endothelial swelling, mesangiolysis, double contours of the glomerular basement membrane, and fibrinoid necrosis [13]. However, this was contraindicated in our patient due to severe thrombocytopenia, precluding a definitive histopathological diagnosis. While genetic testing for complement mutations is not routinely performed in drug-induced TMA, our findings suggest that it may help identify at-risk patients who could benefit from closer monitoring or preemptive intervention. 

## Conclusions

This case underscores carfilzomib-associated TMA as a rare but serious complication in a patient with MM. The rapid onset of TMA after a single cycle, in conjunction with a homozygous deletion in the CFHR3-CFGR1 gene, suggests a possible interaction between drug-induced endothelial injury and underlying complement pathway dysregulation. In this case, the use of eculizumab has proven to be a successful treatment.

## References

[1] Yee AJ (2021). The role of carfilzomib in relapsed/refractory multiple myeloma. Ther Adv Hematol.

[2] João C, Bergantim R, Santos J (2023). Multiple myeloma treatment guidelines by the Portuguese group of multiple myeloma (Article in Portugese). Acta Med Port.

[3] Yui JC, Van Keer J, Weiss BM (2016). Proteasome inhibitor associated thrombotic microangiopathy. Am J Hematol.

[4] Joseph A, Harel S, Mesnard L (2024). Carfilzomib-associated thrombotic microangiopathy: clinical features and outcomes. Nephrol Dial Transplant.

[5] Catanese L, Link K, Rupprecht H (2023). Microangiopathy in multiple myeloma: a case of carfilzomib-induced secondary thrombotic microangiopathy successfully treated with plasma exchange and complement inhibition. BMC Nephrol.

[6] George JN, Nester CM (2014). Syndromes of thrombotic microangiopathy. N Engl J Med.

[7] Gavriilaki E, Anagnostopoulos A, Mastellos DC (2019). Complement in thrombotic microangiopathies: unraveling Ariadne's thread into the labyrinth of complement Therapeutics. Front Immunol.

[8] Genest DS, Patriquin CJ, Licht C, John R, Reich HN (2023). Renal thrombotic microangiopathy: a review. Am J Kidney Dis.

[9] Portuguese AJ, Gleber C, Passero FC Jr, Lipe B (2019). A review of thrombotic microangiopathies in multiple myeloma. Leuk Res.

[10] Fakhouri F, Zuber J, Frémeaux-Bacchi V (2017). Haemolytic uraemic syndrome. Lancet.

[11] Reese JA, Bougie DW, Curtis BR, Terrell DR, Vesely SK, Aster RH, George JN (2015). Drug-induced thrombotic microangiopathy: experience of the Oklahoma Registry and the BloodCenter of Wisconsin. Am J Hematol.

[12] Palomo M, Blasco M, Molina P (2019). Complement activation and thrombotic microangiopathies. Clin J Am Soc Nephrol.

[13] Pallotti F, Queffeulou C, Bellal M (2022). Carfilzomib-induced thrombotic microangiopathy treated with eculizumab: a case report and rapid literature review. Kidney Dial.

